# Supplementary material for: Genomic Epidemiology of SARS-CoV-2 in Tocantins State and the Diffusion of P.1.7 and AY.99.2 Lineages in Brazil
Source: Viruses. 2022 Mar 23;14(4):659. doi: 10.3390/v14040659 (PMC9031820; doi:10.3390/v14040659)
Supplement: Supplementary file 1 [file viruses-14-00659-s001.zip › viruses-1629182-supplementary.pdf]

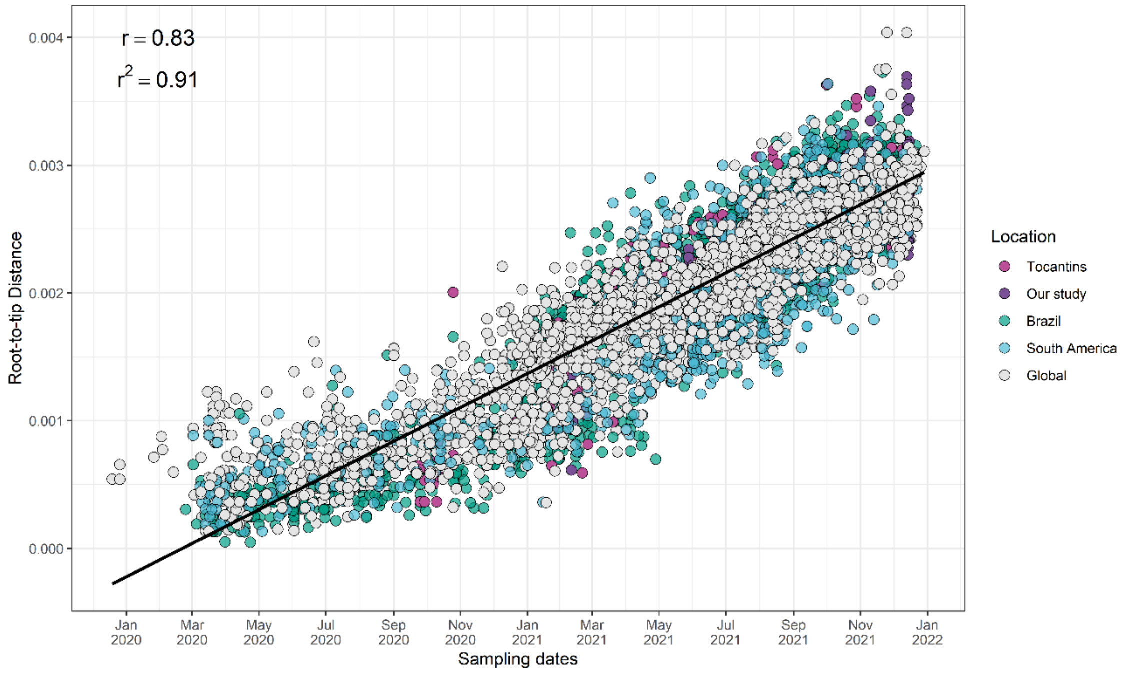

Figure S1. Root-to-tip regression of genetic distances and sampling dates for 9,493 sequences in the final dataset. Correlation coefficient (r) and r squared are depicted above the graph.

Table S1. Epidemiological characteristics of the 241 sequenced samples from Tocantins state.

Study ID	gisaid_epi_isl	Cycle Threshold	Pango Lineage	date	City	Age	Gender
200857	EPI_ISL_7261181	15.5	P.2	2020-12-21	Araguaína	26	Female
211673	EPI_ISL_7260022	17.3	P.2	2021-01-26	Araguaína	21	Male
210150	EPI_ISL_7260679	22.4	P.1	2021-05-05	Araguaína	30	Male
213920	EPI_ISL_7261608	22.6	P.1.1	2021-05-20	Araguaína	28	Female
213924	EPI_ISL_7261616	19.6	P.1	2021-05-20	Araguaína	33	Male
214168	EPI_ISL_7261615	21.7	P.1	2021-05-21	Araguaína	50	Male
361617	EPI_ISL_3602961	17.8	P.1.7	2021-05-26	Gurupi	18	Male
391615	EPI_ISL_3602957	17.1	P.1	2021-05-26	Porto Nacional	51	Female
421614	EPI_ISL_3587057	17.0	P.1.7	2021-05-26	Porto Nacional	56	Male
61604	EPI_ISL_3584949	12.5	P.1	2021-05-26	Formoso do Araguaia	27	Female
101616	EPI_ISL_3602959	18.8	P.1	2021-05-27	Jaú do Tocantins	57	Male
251614	EPI_ISL_3585952	19.7	P.1	2021-05-27	Palmas	67	Female
271614	EPI_ISL_3586500	20.0	P.1.7	2021-05-27	Palmas	44	Female
571615	EPI_ISL_3602958	11.2	P.1	2021-05-27	Porto Nacional	24	Female
601616	EPI_ISL_3602960	18.5	P.1	2021-05-27	Gurupi	28	Male
691614	EPI_ISL_3587490	20.0	P.1	2021-05-27	Palmas	68	Male
711618	EPI_ISL_3610172	17.8	P.1.7	2021-05-27	Rio dos Bois	1	Female
721614	EPI_ISL_3598262	19.3	P.1	2021-05-27	Palmas	42	Female
881618	EPI_ISL_3610352	17.5	P.1.7	2021-05-27	Fortaleza do Tabocão	34	Male
91614	EPI_ISL_3585297	19.3	P.1	2021-05-27	Palmas	51	Female
911617	EPI_ISL_3602964	18.4	P.1	2021-05-27	Lagoa da Confusão	45	Female
941614	EPI_ISL_3602956	19.9	P.1	2021-05-27	Palmas	42	Male
411671	EPI_ISL_3610353	19.9	P.1	2021-05-31	Novo Alegre	12	Female
681672	EPI_ISL_3610357	16.4	P.1.7	2021-05-31	Paraiso do Tocantins	22	Female
771674	EPI_ISL_3610358	19.4	P.1	2021-05-31	Rio Sono	26	Male
101672	EPI_ISL_3610355	19.1	P.1	2021-06-01	Palmas	41	Female
291672	EPI_ISL_3610356	19.9	P.1	2021-06-01	Palmas	16	Male
51674	EPI_ISL_3426438	19.3	P.1	2021-06-01	Palmas	80	Male
731671	EPI_ISL_3610354	18.0	P.1	2021-06-01	Palmas	45	Female
791675	EPI_ISL_3610359	17.7	P.1	2021-06-01	Brejinho de Nazaré	52	Male
512014	EPI_ISL_6677481	18.0	P.1.7	2021-06-26	Gurupi	27	Male
792014	EPI_ISL_6677505	18.4	P.1.7	2021-06-26	Gurupi	39	Male
662015	EPI_ISL_6678406	12.6	P.1.7	2021-06-27	Gurupi	48	Male
922014	EPI_ISL_6678401	18.0	P.1	2021-06-27	Gurupi	57	Male
22013	EPI_ISL_6677480	17.0	P.1.7	2021-06-28	Porto Nacional	26	Female
692025	EPI_ISL_6679928	18.9	P.1.7	2021-06-28	Gurupi	54	Female
722025	EPI_ISL_6681050	17.9	P.1.7	2021-06-28	Gurupi	54	Male
772025	EPI_ISL_6681796	17.2	P.1.7	2021-06-28	Gurupi	86	Female
82014	EPI_ISL_6677590	20.8	P.1.7	2021-06-28	Rio dos Bois	65	Male
862026	EPI_ISL_6683276	18.2	P.1.7	2021-06-28	Fátima	33	Male
132025	EPI_ISL_6678511	18.3	P.1.7	2021-06-29	Gurupi	55	Female
152030	EPI_ISL_6685073	18.7	P.1.7	2021-06-29	Palmas	38	Male
332024	EPI_ISL_6678411	19.6	P.1	2021-06-29	Palmas	50	Male

Study ID	gisaid_epi_isl	Cycle Threshold	Pango Lineage	date	City	Age	Gender
482043	EPI_ISL_7235914	17.7	P.1	2021-06-29	Porto Nacional	49	Male
52030	EPI_ISL_6685077	19.0	P.1.7	2021-06-29	Alvorada	40	Female
622026	EPI_ISL_6682191	18.5	P.1.7	2021-06-29	Gurupi	26	Female
932024	EPI_ISL_6677476	18.2	P.1.7	2021-06-29	Gurupi	22	Male
211511	EPI_ISL_7261516	21.0	P.1	2021-06-30	Santa Tereza do Tocantins	58	Male
232043	EPI_ISL_6686428	18.9	P.1	2021-06-30	Palmeirópolis	47	Female
32043	EPI_ISL_7235716	19.7	P.1	2021-07-01	Dois Irmãos do Tocantins	9	Female
312060	EPI_ISL_7246824	17.4	P.1.7	2021-07-01	Gurupi	52	Male
422060	EPI_ISL_7247859	17.5	P.1	2021-07-01	Paraiso do Tocantins	41	Male
492040	EPI_ISL_6685140	19.2	P.1	2021-07-01	Palmas	5	Female
632046	EPI_ISL_7237031	20.8	P.1	2021-07-01	Gurupi	20	Female
72043	EPI_ISL_7236461	19.2	P.1	2021-07-01	Miranorte	44	Female
852046	EPI_ISL_7243779	18.8	P.1.7	2021-07-01	Gurupi	27	Female
132058	EPI_ISL_7244508	17.4	P.1.7	2021-07-02	Alvorada	66	Female
572061	EPI_ISL_7252530	18.9	P.1.7	2021-07-02	Gurupi	16	Male
872060	EPI_ISL_7258225	18.0	P.1.7	2021-07-02	Gurupi	11	Male
802062	EPI_ISL_7257409	18.1	P.1.7	2021-07-03	Palmas	23	Female
92063	EPI_ISL_7258784	18.1	P.1	2021-07-03	Porto Nacional	29	Female
222087	EPI_ISL_7248142	16.3	P.1.7	2021-07-06	Dois Irmãos do Tocantins	69	Male
612087	EPI_ISL_7248428	17.4	P.1.7	2021-07-06	Araguacema	67	Female
652090	EPI_ISL_7252071	16.0	P.1	2021-07-06	Paraiso do Tocantins	41	Female
932089	EPI_ISL_7249880	17.0	P.1	2021-07-06	Palmeirópolis	42	Male
732089	EPI_ISL_7249088	17.0	P.1.7	2021-07-07	Palmas	37	Female
332180	EPI_ISL_7328333	19.2	P.1.7	2021-07-16	Araguaína	3	Male
352180	EPI_ISL_7317425	17.1	P.1.7	2021-07-16	Araguaína	65	Female
62180	EPI_ISL_7317119	20.1	P.1.7	2021-07-16	Araguaína	48	Male
792180	EPI_ISL_7328334	19.3	P.1	2021-07-16	Filadelfia	34	Male
822179	EPI_ISL_7320262	20.9	P.1.7	2021-07-16	Araguaína	60	Male
912181	EPI_ISL_7359069	18.3	P.1.7	2021-07-16	Araguaína	51	Female
272201	EPI_ISL_7328336	20.6	P.1.7	2021-07-18	Combinado	52	Female
912192	EPI_ISL_7316566	20.5	P.1.7	2021-07-18	Gurupi	79	Male
12205	EPI_ISL_7359183	21.4	P.1.7	2021-07-19	Gurupi	23	Female
222205	EPI_ISL_7359166	21.9	P.1	2021-07-19	Gurupi	43	Female
372204	EPI_ISL_7359144	22.2	P.1.7	2021-07-19	Gurupi	30	Female
382201	EPI_ISL_7328335	19.5	P.1.7	2021-07-19	Combinado	20	Female
552193	EPI_ISL_7317865	22.6	P.1.7	2021-07-19	Formoso do Araguaia	27	Male
62191	EPI_ISL_7314408	22.4	P.1	2021-07-19	Porto Nacional	54	Female
602191	EPI_ISL_7314503	22.1	P.1.7	2021-07-19	Palmas	60	Female
602193	EPI_ISL_7318589	19.9	P.1	2021-07-19	Formoso do Araguaia	61	Male
632195	EPI_ISL_7319537	22.7	P.1	2021-07-19	Dois Irmãos do Tocantins	36	Female
662194	EPI_ISL_7319006	23.0	P.1.7	2021-07-19	Aurora do Tocantins	32	Male
682195	EPI_ISL_7319775	22.7	P.1.7	2021-07-19	Santa Maria do Tocantins	46	Female
102203	EPI_ISL_7328337	22.2	P.1	2021-07-20	Palmas	62	Female
152204	EPI_ISL_7314272	20.3	P.1.7	2021-07-20	Conceição do Tocantins	69	Male
372203	EPI_ISL_7359155	21.1	P.1	2021-07-20	Palmas	22	Male

Study ID	gisaid_epi_isl	Cycle Threshold	Pango Lineage	date	City	Age	Gender
382213	EPI_ISL_7359135	20.8	P.1.7	2021-07-20	Araguaína	41	Female
552213	EPI_ISL_7314321	20.0	P.1.7	2021-07-20	Araguaína	22	Female
602213	EPI_ISL_7359117	17.7	P.1.7	2021-07-20	Araguaína	28	Male
732203	EPI_ISL_7359094	22.6	P.1	2021-07-20	Rio Sono	42	Male
842203	EPI_ISL_7359083	17.7	P.1	2021-07-20	Lagoa da Confusão	34	Male
902203	EPI_ISL_7359075	20.4	P.1	2021-07-20	Lagoa da Confusão	9	Female
432250	EPI_ISL_7615307	23.5	P.1	2021-07-23	Abreulândia	62	Female
442250	EPI_ISL_7615308	24.2	P.1	2021-07-23	Araguacema	65	Female
52234	EPI_ISL_7615309	20.4	P.1	2021-07-23	Palmas	29	Male
432247	EPI_ISL_7615310	22.8	P.1	2021-07-24	Palmas	34	Male
632248	EPI_ISL_7615332	24.2	P.1	2021-07-24	Palmas	38	Male
632321	EPI_ISL_7359106	17.1	P.1.7	2021-08-01	Combinado	33	Female
642321	EPI_ISL_7359100	20.2	P.1.7	2021-08-01	Palmas	36	Male
212320	EPI_ISL_7315509	19.3	P.1.7	2021-08-02	Paraiso do Tocantins	80	Male
102321	EPI_ISL_7359176	22.7	P.1.7	2021-08-03	Palmas	45	Male
112322	EPI_ISL_7314566	20.9	P.1.7	2021-08-03	Miracema do Tocantins	49	Female
302320	EPI_ISL_7316019	20.2	P.1.7	2021-08-03	Palmas	46	Male
522321	EPI_ISL_7359128	19.2	P.1	2021-08-03	Palmas	53	Female
932321	EPI_ISL_7359060	14.3	P.1.7	2021-08-03	Combinado	16	Female
502342	EPI_ISL_7615305	15.0	AY.42	2021-08-05	Taguatinga	62	Female
512342	EPI_ISL_7615306	14.7	AY.42	2021-08-05	Taguatinga	71	Male
832342	EPI_ISL_7615311	16.7	P.1.7	2021-08-05	Gurupi	69	Male
852342	EPI_ISL_7615312	18.8	P.1.7	2021-08-05	Gurupi	52	Female
862343	EPI_ISL_7615313	16.7	P.1.7	2021-08-05	Gurupi	47	Male
902355	EPI_ISL_7615333	26.8	P.1.7	2021-08-08	Palmas	53	Female
392383	EPI_ISL_7615317	17.9	P.1	2021-08-11	Alvorada	61	Female
552384	EPI_ISL_7615319	16.4	P.1.7	2021-08-11	Fátima	38	Female
752383	EPI_ISL_7615318	17.4	P.1.7	2021-08-11	Araguacema	17	Male
812382	EPI_ISL_7615315	16.3	P.1.7	2021-08-11	Palmeirópolis	19	Female
902381	EPI_ISL_7615314	15.6	P.1	2021-08-11	Gurupi	34	Female
932382	EPI_ISL_7615316	16.1	P.1.7	2021-08-11	Sandolândia	69	Male
692404	EPI_ISL_7615320	19.4	P.1	2021-08-14	Natividade	41	Male
502419	EPI_ISL_7615321	17.2	P.1.7	2021-08-16	Figueirópolis	34	Female
512419	EPI_ISL_7615322	17.9	P.1.7	2021-08-16	Figueirópolis	9	Male
532419	EPI_ISL_7615323	17.1	P.1.7	2021-08-16	Figueirópolis	35	Female
822409	EPI_ISL_7615331	22.5	P.1	2021-08-16	Palmas	26	Male
872409	EPI_ISL_7615334	18.5	P.1.7	2021-08-16	Lavandeira	43	Female
182419	EPI_ISL_7615330	18.5	P.1.7	2021-08-17	Formoso do Araguaia	33	Female
192419	EPI_ISL_7615328	16.3	P.1.7	2021-08-17	Formoso do Araguaia	15	Female
242419	EPI_ISL_7615324	18.7	P.1.7	2021-08-17	Formoso do Araguaia	70	Female
302419	EPI_ISL_7615329	18.4	P.1.7	2021-08-17	Formoso do Araguaia	38	Female
212441	EPI_ISL_7657567	19.2	AY.99.2	2021-08-20	Palmas	28	Male
602450	EPI_ISL_7615325	21.2	P.1.7	2021-08-21	Palmas	46	Male
22451	EPI_ISL_7615326	22.1	P.1	2021-08-22	Bom Jesus do Tocantins	34	Male
332454	EPI_ISL_7615327	18.4	P.1.7	2021-08-22	Gurupi	69	Female

Study ID	gisaid_epi_isl	Cycle Threshold	Pango Lineage	date	City	Age	Gender
522472	EPI_ISL_7657568	23.0	AY.99.2	2021-08-25	Palmas	40	Female
482479	EPI_ISL_7657565	18.0	AY.99.2	2021-08-26	Palmas	26	Female
472494	EPI_ISL_7657566	19.6	AY.99.2	2021-08-28	Porto Nacional	82	Male
622492	EPI_ISL_7657581	24.5	AY.99.2	2021-08-28	Palmas	28	Male
212500	EPI_ISL_7657582	19.5	AY.99.2	2021-08-30	Palmas	17	Male
42507	EPI_ISL_7657569	17.5	AY.99.2	2021-08-30	Palmas	73	Female
662520	EPI_ISL_7657574	21.2	P.1.7	2021-09-01	Paranã	32	Male
672520	EPI_ISL_7657591	23.1	P.1.7	2021-09-01	Paranã	21	Male
712520	EPI_ISL_7657575	21.0	P.1.14	2021-09-01	Paranã	39	Female
752520	EPI_ISL_7657576	23.9	P.1.7	2021-09-01	Paranã	63	Female
762520	EPI_ISL_7657578	18.6	P.1	2021-09-01	Paranã	50	Male
772520	EPI_ISL_7657577	22.0	P.1.7	2021-09-01	Paranã	36	Male
32526	EPI_ISL_7657592	20.3	AY.99.2	2021-09-02	Palmas	44	Female
112529	EPI_ISL_7657580	20.0	P.1.7	2021-09-03	Palmas	53	Male
892567	EPI_ISL_7657559	20.9	AY.99.2	2021-09-12	Santa Rosa do Tocantins	33	Male
352570	EPI_ISL_7657563	16.9	AY.99.2	2021-09-13	Porto Nacional	13	Female
362570	EPI_ISL_7657564	16.5	AY.99.2	2021-09-13	Porto Nacional	46	Female
702564	EPI_ISL_7657558	20.5	AY.99.2	2021-09-13	Palmas	18	Female
142569	EPI_ISL_7657561	17.4	AY.122	2021-09-14	Araguatins	24	Male
32570	EPI_ISL_7657562	18.1	AY.99.2	2021-09-14	Palmas	31	Female
622570	EPI_ISL_7657579	19.7	P.1.7	2021-09-14	Palmas	23	Female
772568	EPI_ISL_7657560	18.6	AY.116	2021-09-14	Palmas	56	Male
292578	EPI_ISL_7657570	18.3	AY.99.2	2021-09-15	Santa Maria do Tocantins	2	Male
332577	EPI_ISL_7657572	19.7	AY.99.2	2021-09-15	Palmas	15	Female
322600	EPI_ISL_7657585	18.3	AY.99.2	2021-09-20	Pindorama do Tocantins	9	Male
332600	EPI_ISL_7657587	18.5	AY.99.2	2021-09-20	Pindorama do Tocantins	7	Male
872600	EPI_ISL_7657573	19.2	AY.43	2021-09-20	Palmas	25	Male
892600	EPI_ISL_7657590	19.9	AY.99.2	2021-09-20	Palmas	8	Female
782598	EPI_ISL_7657571	16.6	AY.42	2021-09-21	Lagoa da Confusão	38	Male
413146	EPI_ISL_7661119	21	AY.99.2	2021-10-13	Palmas	39	Male
413250	EPI_ISL_7661128	16.7	AY.43	2021-10-18	Palmas	35	Male
42807	EPI_ISL_7661120	19.2	AY.99.2	2021-11-04	Palmas	25	Male
513886	EPI_ISL_7661129	18	AY.99.2	2021-11-16	Palmas	38	Female
913216	EPI_ISL_7661121	19.7	AY.43	2021-10-15	Palmas	1 month	Male
1013129	EPI_ISL_7661130	21.9	AY.99.2	2021-10-11	Palmas	65	Male
1013130	EPI_ISL_7661144	25.8	AY.99.2	2021-10-11	Palmas	78	Male
1013132	EPI_ISL_7661145	22.1	AY.99.2	2021-10-12	Palmas	18 days	Male
1013162	EPI_ISL_7661122	16.5	AY.99.2	2021-10-14	Palmas	84	Female
1113216	EPI_ISL_7661146	17.6	AY.99.2	2021-10-15	Abreulândia	42	Female
1113256	EPI_ISL_7661123	21.1	AY.43	2021-10-18	Palmas	55	Male
1413055	EPI_ISL_7661150	19.5	AY.99.2	2021-10-07	Porto Nacional	74	Female
1413062	EPI_ISL_7661141	26	AY.99.2	2021-10-07	Formoso do Araguaia	43	Female
1413216	EPI_ISL_7661140	25.9	AY.99.2	2021-10-15	Palmas	55	Male
162807	EPI_ISL_7661131	21.1	AY.99.2	2021-10-15	Palmas	19	Female
232839	EPI_ISL_7661124	19.9	AY.99.2	2021-11-11	Palmas	45	Female

Study ID	gisaid_epi_isl	Cycle Threshold	Pango Lineage	date	City	Age	Gender
362839	EPI_ISL_7661125	18.1	AY.116	2021-11-11	Palmas	40	Female
382807	EPI_ISL_7661126	18.2	AY.43	2021-11-04	Palmas	26	Male
492807	EPI_ISL_7661142	18.2	AY.99.2	2021-11-04	Palmas	40	Male
582839	EPI_ISL_7661127	18.4	AY.99.2	2021-11-11	Palmas	22	Male
592807	EPI_ISL_7661151	17.3	AY.99.2	2021-11-04	Palmas	36	Male
712876	EPI_ISL_7661132	22	AY.99.2	2021-11-20	Rio dos Bois	27	Female
872839	EPI_ISL_7661133	15.5	AY.99.2	2021-11-11	Palmas	49	Female
2100421	EPI_ISL_7661147	21.4	AY.99.2	2021-10-21	Piraquê	46	Female
2100096	EPI_ISL_7661134	19.9	AY.43	2021-10-20	São Miguel do Tocantins	61	Male
2103932	EPI_ISL_7661149	18.7	AY.99.2	2021-10-20	Araguaína	32	Male
2103934	EPI_ISL_7661135	19.4	AY.99.2	2021-10-20	Araguaína	83	Male
2103333	EPI_ISL_7661136	20.3	AY.43	2021-10-21	Araguaína	20	Male
2100775	EPI_ISL_7661137	22.0	AY.3	2021-10-20	Couto Magalhães	32	Male
2100420	EPI_ISL_7661148	16.1	AY.99.2	2021-10-20	Piraquê	24	Female
2101977	EPI_ISL_7661138	17.6	AY.43	2021-10-20	Augustinópolis	36	Female
2101149	EPI_ISL_7661143	17.0	AY.99.2	2021-10-20	Araguaína	71	Male
2106665	EPI_ISL_7661139	19.1	AY.99.2	2021-10-20	Colinas do Tocantins	42	Female
413009	EPI_ISL_10083514	17.2	AY.99.2	2021-12-13	Palmas	9	Female
583009	EPI_ISL_10083515	21	AY.99.2	2021-12-13	Palmas	39	Female
873009	EPI_ISL_10083516	19.3	AY.43	2021-12-13	Palmas	32	Male
13008	EPI_ISL_10083517	18.9	AY.43	2021-12-10	Porto Nacional	29	Male
273008	EPI_ISL_10083518	21.7	AY.99.2	2021-12-13	Gurupi	70	Female
353008	EPI_ISL_10083519	20.4	AY.43	2021-12-11	Gurupi	55	Female
813008	EPI_ISL_10083520	20.9	AY.43	2021-12-13	Cariri do Tocantins	36	Female
853008	EPI_ISL_10083521	21.3	AY.43	2021-12-13	Cariri do Tocantins	30	Female
203007	EPI_ISL_10083522	19.7	AY.99.2	2021-12-13	Palmas	24	Female
213007	EPI_ISL_10083523	17.9	AY.99.2	2021-12-13	Gurupi	32	Female
443007	EPI_ISL_10083524	20.6	AY.99.2	2021-12-13	Palmas	42	Female
573007	EPI_ISL_10083525	23.3	AY.99.2	2021-12-13	Palmas	41	Male
653007	EPI_ISL_10083526	16.9	AY.43	2021-12-10	Figueirópolis	62	Male
633007	EPI_ISL_8170818	19.8	BA.1	2021-12-10	Gurupi	29	Male
673007	EPI_ISL_10083527	19.8	AY.99.2	2021-12-10	Gurupi	25	Male
473014	EPI_ISL_10083528	16.3	AY.99.2	2021-12-13	Palmas	57	Male
523014	EPI_ISL_10083529	18.2	AY.99.2	2021-12-13	Porto Nacional	72	Female
533014	EPI_ISL_10083530	18.9	AY.99.2	2021-12-13	Porto Nacional	43	Male
603014	EPI_ISL_10083531	21.1	AY.99.2	2021-12-13	Porto Nacional	50	Male
693014	EPI_ISL_10083532	12.7	AY.99.2	2021-12-13	Miracema do Tocantins	39	Female
723014	EPI_ISL_10083533	18.9	AY.99.2	2021-12-13	Miracema do Tocantins	57	Female
763014	EPI_ISL_10083534	18	AY.43	2021-12-13	Miracema do Tocantins	35	Female
823014	EPI_ISL_10083535	20.6	AY.99.2	2021-12-13	Miracema do Tocantins	9	Female
873014	EPI_ISL_10083536	21.8	AY.43	2021-12-13	Miracema do Tocantins	49	Female
323017	EPI_ISL_10083537	14.8	AY.43	2021-12-13	Porto Nacional	66	Female
663017	EPI_ISL_10083538	17.5	AY.43	2021-12-14	Palmas	44	Female
733017	EPI_ISL_10083539	22.3	AY.43	2021-12-14	Palmas	63	Female
813017	EPI_ISL_10083540	22.7	AY.43	2021-12-14	Palmas	23	Female

Study ID	gisaid_epi_isl	Cycle Threshold	Pango Lineage	date	City	Age	Gender
893017	EPI_ISL_10083541	17.6	AY.43	2021-12-14	Palmas	61	Female
53018	EPI_ISL_10083542	21.6	AY.43	2021-12-14	Palmas	59	Female
203018	EPI_ISL_10083543	18.8	AY.99.2	2021-12-13	Palmas	45	Male
253018	EPI_ISL_10083544	21.7	AY.99.2	2021-12-13	Palmas	28	Male
293018	EPI_ISL_10083545	19.8	AY.99.2	2021-12-13	Brejinho de Nazaré	49	Male
313018	EPI_ISL_10083546	18.4	AY.43	2021-12-13	Brejinho de Nazaré	38	Female
333018	EPI_ISL_10083547	20.5	AY.99.2	2021-12-13	Brejinho de Nazaré	45	Female
693018	EPI_ISL_10083548	19.1	AY.99.2	2021-12-14	Palmas	50	Female
893018	EPI_ISL_10083549	16.3	AY.43	2021-12-14	Palmas	18	Female
813019	EPI_ISL_10083550	19.5	AY.34.1.1	2021-12-14	Palmas	16	Female
373019	EPI_ISL_10083551	20.3	AY.99.2	2021-12-14	Tocantinia	36	Female
103039	EPI_ISL_10083552	20.5	AY.43	2021-12-15	Tupiratins	22	Male
223039	EPI_ISL_10083553	17.9	AY.99.2	2021-12-16	Santa Tereza do Tocantins	41	Male
383039	EPI_ISL_10083554	19.8	AY.43	2021-12-16	Palmas	66	Male
463039	EPI_ISL_10083555	22.9	AY.99.2	2021-12-16	Dianapolis	55	Female
613039	EPI_ISL_10083556	22.3	AY.43	2021-12-16	Palmas	25	Male
673039	EPI_ISL_10083557	18.7	AY.99.2	2021-12-16	Palmas	41	Male
803039	EPI_ISL_10083558	16.3	AY.99.2	2021-12-16	Palmas	34	Male

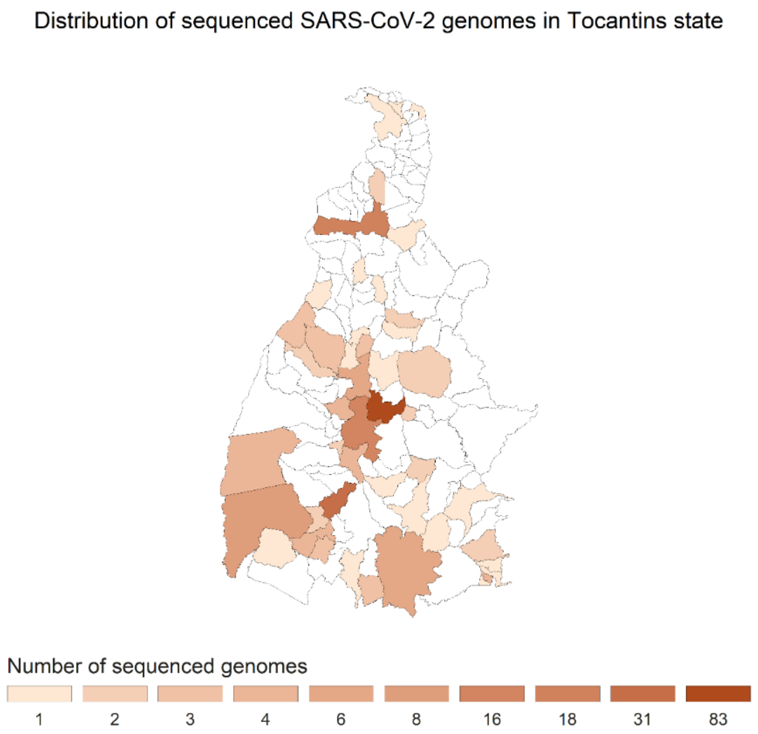

Figure S2. Spatiotemporal distribution of the 241 sequenced genomes from Tocantins state. The figure was generated using R v4.1.2 with the following packages: ggplot2, geojsonio, sf, broom and dplyr.

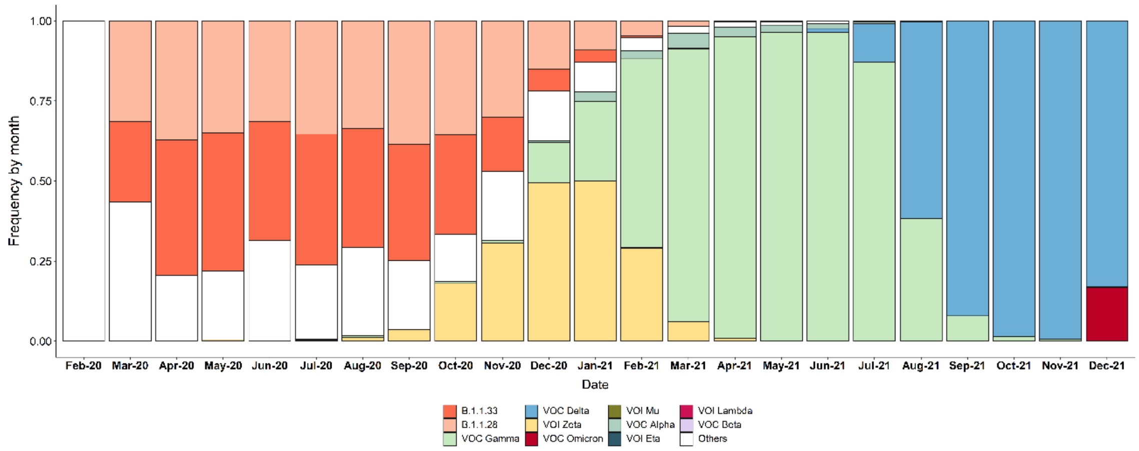

Figure S3. Frequency of SARS-CoV-2 variants in Brazil from February 2020 to December 2021. The figure was generated based on 91,201 Brazilian genomes deposited on GISAID up to 31 December 2021. For B.1.1.33 and B.1.1.28 was used Pango nomenclature and per the latest WHO nomenclature of SARS-CoV-2 variants.
